# Word Familiarity Modulated the Effects of Category Familiarity on Memory Performance

**DOI:** 10.3389/fpsyg.2018.01429

**Published:** 2018-08-08

**Authors:** Xueling Ning, Cuihong Li, Jiongjiong Yang

**Affiliations:** School of Psychological and Cognitive Sciences, Beijing Key Laboratory of Behavior and Mental Health, Peking University, Beijing, China

**Keywords:** episodic memory, recognition, prior knowledge, word familiarity, category familiarity

## Abstract

Previous studies have shown that prior knowledge can have both enhancing and detrimental effects on memory for relevant information. Few studies have explored the boundary conditions under which prior knowledge facilitates or interferes with memory processes. In addition, to what extent the effects of prior knowledge change over time is unclear. In this study, we addressed this question by separating category familiarity (i.e., prior conceptual knowledge) and stimulus familiarity at different retention intervals. Participants were tested with a recognition task after they learned four types of words, that is., familiar words from familiar categories (FwordFcate) and unfamiliar categories (FwordUcate) as well as unfamiliar words from familiar (UwordFcate) and unfamiliar categories (UwordUcate). The results showed a significant interaction between category familiarity and word familiarity, that is, unfamiliar words, but not familiar words, from familiar categories were remembered better than those from unfamiliar categories. The enhancing effect of category familiarity depended on the recollection process and remained stable over time. This study suggested that stimulus familiarity modulates the effects of category familiarity on memory performance, and clarified the boundary conditions for the effects of prior knowledge.

## Introduction

Generally, we more easily remember events that are familiar or are relevant to our prior knowledge. Many studies have suggested that information that involves prior knowledge is more easily remembered than completely new information. People benefit from integration of the newly acquired information with pre-existing knowledge within the relevant area. For example, memory performance was higher after participants learned familiar (vs. unfamiliar) essays (Bransford and Johnson, 1972) or after they viewed the first half of a film 1 day before a memory test (van Kesteren et al., 2010). Students with background knowledge of biology or education had better memories for sentences that described new facts in their familiar field (Van Kesteren et al., 2014). Brandt et al. (2005) enrolled participants in the domain of radiography and psychology, and asked them to learn words in the two domains. The results showed that memory performance was higher for familiar than unfamiliar academic backgrounds, and this effect was attributable to recollection rather than familiarity process. The effect of prior knowledge, or the congruent effect, has been found when different experimental manipulations were used, such as category knowledge (e.g., DeWitt et al., 2012; Hennies et al., 2016), academic knowledge (e.g., Brandt et al., 2005; Van Kesteren et al., 2014), Star Trek knowledge (Long and Prat, 2002), and football knowledge (Rawson and Van Overschelde, 2008).

However, other studies have suggested that prior knowledge has the opposite effect on memory performance, showing that information with prior knowledge is remembered worse than that without prior knowledge. For example, in a study by Davis et al. (2012), participants were asked to examine beetle pictures; some of the images had prototypical beetle features, while others did not. The results showed that the pictures that were not congruent with their beetle knowledge were remembered better than the congruent ones. Sweegers et al. (2014) trained participants to memorize face-house pairs based on specific rules. The next day, they learned rule-congruent or incongruent face-house pairs. The results showed that memories of faces and face-house associations were much better in the incongruent condition than in the congruent condition. In addition, this incongruent effect is vivid (Neuschatz et al., 2002; Yamada and Itsukushima, 2013) and leads to a higher false alarm rate in recognition tasks (e.g., Neuschatz et al., 2002; De Brigard et al., 2017). The opposite effects of prior knowledge, or incongruent effects, are also reported for testing memory of actions or objects in a situation occurring within a videotaped lecture (e.g., Neuschatz et al., 2002), a kitchen (e.g., Yamada and Itsukushima, 2013), or a bathroom (e.g., Kuppers and Bayen, 2014).

There are various models to explain these congruent and incongruent effects. For the congruent effect, the models assume that information congruent with prior knowledge can attract more attentional resources (Rawson and Van Overschelde, 2008; DeWitt et al., 2012), and can be easily assimilated into an existing knowledge system (Alba and Hasher, 1983; see also Van Kesteren et al., 2012). For the incongruent effect, the models emphasize the role of distinctive features of incongruent stimuli. For example, the schema copy and tag models propose that recognition memory should be better for irrelevant than for relevant stimuli, because the former are separately and distinctively tagged in the memory trace (e.g., Schank and Abelson, 1977; Nakamura et al., 1985). Distinctive features are associated with a unique encoding context (Tulving and Kroll, 1995; Kormi-Nouri et al., 2005), and could attract more attention-related resources (Brewer and Treyens, 1981), leading to higher memory performance for the incongruent than congruent stimuli.

Thus far, few studies have explored the boundary conditions under which prior knowledge facilitates or interferes with memory processes and its mechanisms. We consider that the difference between these two types of results reflects the difference between category familiarity and stimuli familiarity. For the congruent effect, memory for new/unfamiliar stimuli from familiar and unfamiliar categories (e.g., sentences for new words in a familiar or unfamiliar knowledge background) was tested (e.g., Brandt et al., 2005; DeWitt et al., 2012; Van Kesteren et al., 2014; Hennies et al., 2016). The effect of prior knowledge is thus referred to as the difference between familiar and unfamiliar categories. In contrast, for the incongruent effect, memory for familiar or typical stimuli within a familiar category or situation (e.g., beetle pictures) was tested. In many of these studies, the effect of prior knowledge was referred to as the difference between expected and unexpected events/stimuli in a familiar situation (e.g., actions and objects in a kitchen or bathroom) (e.g., Neuschatz et al., 2002; Yamada and Itsukushima, 2013; Kuppers and Bayen, 2014). As the events in different knowledge systems were not compared, the two types of effect of prior knowledge are different. To be clear, the former is referred to as the effect of category familiarity, and the latter is referred to as the stimulus familiarity in this study.

Having some prior conceptual knowledge is different from being familiar with certain exemplars within the prior knowledge system. For example, we may know that a beaver belongs to the familiar four-footed animal category, but we are unfamiliar with features of the beaver (i.e., it is an unfamiliar exemplar from a familiar category). We may also know that a sparrow belongs to the bird category, but we are unable to recognize other birds within the bird category (i.e., it is a familiar exemplar from an unfamiliar category). It is possible that familiarity of the exemplars modulates subsequent effect of category familiarity. Studies have shown that stimulus familiarity itself influences memory performance (Deese, 1959; Gorman, 1961; Tulving and Kroll, 1995). In a study by Tulving and Kroll (1995), participants studied words presented in six repetitions and novel words. Recognition performance was better for novel words than familiar ones. Newly learned information may conflict or be congruent with information in a prior knowledge system (Anderson, 1983), leading to an enhancing or detrimental effect for subsequent memory. Therefore, it is necessary to dissociate the effects of category familiarity and stimulus familiarity to explore how the two factors interact to influence memory.

In addition, we considered the influence of retention interval on the effects of category familiarity and stimulus familiarity. Most studies on the effect of category familiarity tested memory right after encoding or on the next day. This suggested that assimilation of information consistent with prior knowledge into an existing knowledge system can proceed rapidly (Tse et al., 2007; McClelland, 2013). Recent studies have also suggested that sleep facilitates memory consolidation, leading to a slower forgetting rate when the information is consistent with prior knowledge (Durrant et al., 2015; Hennies et al., 2016). However, as few studies have focused on time changes in the effect of category familiarity other than 1 day, less is known about whether the effect would remain for a longer interval. In addition, the effect of stimulus familiarity is usually obtained right after encoding (e.g., Tulving and Kroll, 1995; Neuschatz et al., 2002), and to what extent the effect of stimulus familiarity changes with the passage of time is unclear.

Therefore, in this study, factors of category familiarity, word familiarity and retention interval were included to investigate their effects on memory performance. We first defined prior conceptual knowledge as knowledge of familiar categories. This manipulation was also applied in some previous studies (e.g., DeWitt et al., 2012; Hennies et al., 2016), as familiar categories provided more conceptual knowledge for participants to use during memory tasks. The effect of prior conceptual knowledge, or effect of category familiarity, refers to the difference between familiar and unfamiliar categories, whereas the effect of stimulus familiarity concerns differences between familiar and unfamiliar exemplars. We then selected familiar and unfamiliar categories. Category selection was based on standard norms (Battig and Montague, 1969; Van Overschelde et al., 2004), and the selected categories were confirmed by a separate group of participants. Finally, we selected familiar and unfamiliar exemplars from the two types of categories based on standard norms (Van Overschelde et al., 2004). During encoding, participants were required to read each Chinese word and rate its familiarity. During retrieval, they were asked to make an old/new judgment for each word. To test whether the effect of category familiarity lasted for a long time, the participants were tested at four retention intervals. Previous studies have suggested that recollection rather than familiarity process contribute more to the effect of prior knowledge (e.g., Long and Prat, 2002; Brandt et al., 2005). To separate the contributions of recollection and familiarity to the effects of category familiarity and stimulus familiarity over time, we further asked the participants to make a remember/know/guess judgment after the old/new judgment.

We hypothesized that category familiarity and word familiarity would interact with each other to influence the subsequent memory performance. The enhancement effect of category familiarity would occur only for unfamiliar words because familiar words within familiar categories are more subject to interference. Prior conceptual knowledge provides a semantic context for forming more elaborate or distinctive memories and boosting memory performance for unfamiliar words. The effect of word familiarity occurs for familiar categories, showing that unfamiliar words are recognized better than familiar words. As the new information that involves conceptual knowledge can be consolidated quickly (Tse et al., 2007; Moscovitch et al., 2016), we hypothesized that the enhanced effect of category familiarity would remain strong over time. The effect of stimulus familiarity is mainly associated with the distinctiveness of unfamiliar words. When the time goes by, the old familiar words are harder to be distinguished from their distractors than old unfamiliar words, so the effect of word familiarity would appear when the interval is longer.

## Materials and Methods

### Participants

Twenty healthy, right-handed participants (10 males) with a mean age of 21.75 ± 2.69 years were recruited in the study. All of the participants were native Chinese speakers, and they all provided written informed consent in accordance with the procedures and protocols, which were approved by the Review Board of School of Psychological and Cognitive Sciences, Peking University.

### Materials

Three within-subject factors were included in the study: category familiarity (familiar category as Fcate and unfamiliar category as Ucate), stimulus familiarity (familiar words as Fword and unfamiliar word as Uword), and retention interval (10-min, 1-day, 1-week, and 1-month). Chinese words were used in the study. The words were divided into four conditions at each retention interval: familiar words from familiar categories (FwordFcate) and unfamiliar categories (FwordUcate), unfamiliar words from familiar categories (UwordFcate) and unfamiliar categories (UwordUcate).

We first selected 12 familiar (e.g., vegetable) and 12 unfamiliar categories (e.g., insects). Among them, 10 familiar and 9 unfamiliar categories were from Battig and Montague (1969) and Van Overschelde et al. (2004). In the study of Van Overschelde et al. (2004), participants had 30 s to generate as many responses to each category as possible, after which time the next category name was presented. The category potency and category rank are indexes to represent category familiarity. The category potency is computed by dividing the total number of responses given for a category by the total number of participants who gave responses to that category. The rank score is the mean potency for each category; the lower score is, the more familiar the category is. The mean category potencies for the familiar and unfamiliar categories were 6.98 ± 1.80 and 5.88 ± 0.83 [*t*(17) = 1.68, *p* = 0.11], and the mean category ranks for the familiar and unfamiliar categories were 21.30 ± 17.37 and 33.44 ± 13.43 [*t*(17) = 1.69, *p* = 0.11]. Considering the cultural and age difference, we added two familiar (i.e., Chinese food and Chinese daily utensils) and three unfamiliar categories (i.e., merchant brand, Chinese medicine, and Chinese tea).

**FIGURE 1:**
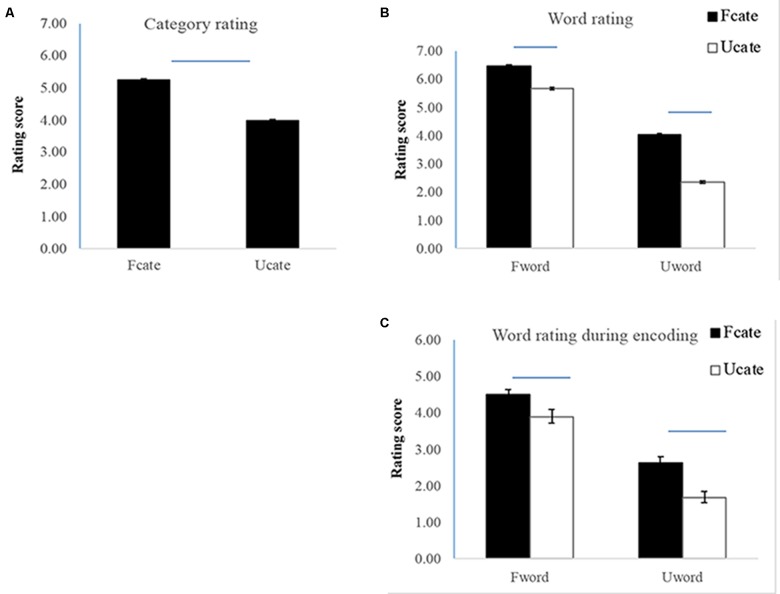
Results of familiarity rating. **(A)** Category familiarity rating. **(B)** Word familiarity rating. **(C)** Word familiarity rating during encoding.

The familiarity of the 24 categories was rated by another 19 participants (13 males, with the mean age of 22.6 ± 2.58 years). For each category, the participants were asked to rate whether they were familiar about its general knowledge (e.g., DeWitt et al., 2012) and could generate many exemplars from it (Battig and Montague, 1969; Van Overschelde et al., 2004) (l for most unfamiliar and 7 for most familiar). The mean rating scores for the familiar and unfamiliar categories were 5.25 ± 0.77 and 3.99 ± 0.73 (**Figure 1A**). The difference was significant, *t*(18) = 16.02, *p* < 0.001. In addition, to confirm that the material selection was consistent with standard norms, we compared the categories that were selected from Battig and Montague (1969) and Van Overschelde et al. (2004), and that were added in our study. For the familiar categories, the mean rating scores for the two sets were 5.46 ± 0.51 and 6.13 ± 0.33, with no significant difference between them, *t*(10) = 1.76, *p* = 0.11. For the unfamiliar categories, the mean rating scores for the two sets were 3.65 ± 0.36 and 3.52 ± 0.34, with no significant difference between them, *t*(10) = 0.55, *p* = 0.59. The results confirmed the category selection is consistent with the standard norms.

We then selected 576 familiar and unfamiliar exemplars within the selected categories from Van Overschelde et al. (2004) and the encyclopedic knowledge websites in the Internet. In the study of Van Overschelde et al. (2004), word familiarity referred to as the proportion of all participants who gave the particular response within a category. The mean word familiarity for the selected exemplars was 0.30 ± 0.24 and 0.15 ± 0.15 for the familiar and unfamiliar words [*t*(148) = 3.46, *p* = 0.001]. As unfamiliar words were few in the norm of Van Overschelde et al. (2004), 40% of the familiar exemplars and 12% of the unfamiliar exemplars were selected from it. The remaining exemplars were selected from the Internet.

To ensure the validity of word selection, word familiarity was rated by the 19 participants who rated the category familiarity. For each word, the participants were asked to rate the extent to which they were familiar with the word (l for most unfamiliar and 7 for most familiar). The word rating scores were 6.06 ± 0.77 and 3.20 ± 0.82 for the familiar and unfamiliar words, respectively, *F*(1,18) = 391.43, *p* < 0.001, $\eta_{p}^{2}$ = 0.96. The words from the familiar categories were rated as more familiar than those from the unfamiliar categories, *F*(1,18) = 230.18, *p* < 0.001, $\eta_{p}^{2}$ = 0.93. The interaction between category familiarity and word familiarity was significant, *F*(1,18) = 19.22, *p* < 0.001, $\eta_{p}^{2}$ = 0.52. The interaction was manifested as larger difference in word familiarity for unfamiliar (vs. familiar) categories, although the word familiarity differences reached significance for both familiar and unfamiliar categories (*p*’s < 0.001) (**Figure 1B**).

In addition, we compared the word familiarity for words that were selected from Van Overschelde et al. (2004) and that were added in our study. For the familiar words, the mean rating scores for the two sets were 6.14 ± 0.50 and 6.07 ± 0.56, with no significant difference between them, *t*(286) = 1.11, *p* = 0.27. For the unfamiliar words, the mean rating scores for the two sets were 3.49 ± 0.92 and 3.10 ± 0.18, with no statistically significant difference between them, *t*(286) = 1.87, *p* = 0.06. The results confirmed that the word selection is consistent with the standard norms.

The 576 words were divided into two sets. Half of the words, which were grouped into one set, were learned during encoding, and the other half were used as new stimuli in the recognition task. For each set, the 288 words were divided into four subsets to be used for four retention intervals (72 words per interval). Within each set, there was the same number of words for the four stimulus types (18 words per type). The words in the eight subsets had comparable word familiarity and numbers of strokes (*p*’s* >* 0.10). The subsets were counterbalanced so that each subset had an equal chance of being used for the four retention intervals as well as for the old/new subsets.

### Procedure

**FIGURE 2:**
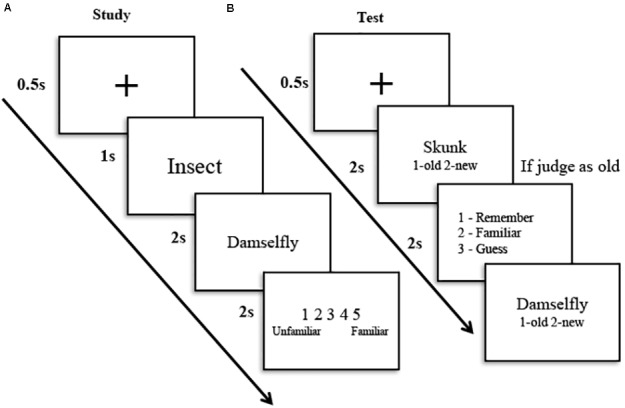
Procedure of the study **(A)** and test **(B)** session. During the study phase, participants were presented with the category name, followed by the word, and were asked to rate the familiarity. During the test phase, participants were asked to make old/new judgment, then if the judgment was old, they continued to make an R/K/G judgment. The Chinese words were translated into English for illustration purpose.

The participants learned 288 words on the same day; then, they performed the recognition tests at four-retention intervals. Each studied item was tested only once. During the study phase (**Figure 2A**), for each trial, the category name of the following word was presented at the center of the screen for 1 s; then, the word was presented for 2 s while the participants were asked to read the word aloud and rate the familiarity of the word (1 for most unfamiliar and 5 for most familiar). The category name was presented so that the participants could process the word with the manipulation of category familiarity (Alba and Hasher, 1983). This also excluded the influence of word transparency in Chinese words. All the words were pseudo-randomly presented during the encoding phase so that no more than three words that were in the same condition were presented consecutively.

During the test phase, each of the 144 words (half old, half new) was presented at the center of the screen for 2 s, and the participants judged whether the word was old or new as accurately and quickly as possible by the keyboard (**Figure 2B**). If the word was judged to be old, it was presented again for 1 s, and the participants were asked to judge whether they remembered, knew, or guessed it. If the participants judged that they could retrieve stimulus-related details or contexts, they responded with a judgment of “remember.” If they only felt that the stimulus was familiar without any detailed information, they responded with a judgment of “know.” If they did not believe that they retrieved the stimulus by the two above mentioned processes, they responded with a judgment of “guess.” The old and new words were pseudo-randomly presented at each retention interval for each participant so that no more than three words in the same condition were presented consecutively. The press button for the recognition judgment was counterbalanced across the participants.

Before each test phase, to avoid a rehearsal from the study phase, the participants were asked to count backward by 7 continuously from 1,000 for 5 min. The participants had separate opportunities to practice study and test trials before the formal phases.

### Data Analysis

Corrected recognition (Hit-FA) was calculated and analyzed using repeated-measures ANOVA with category familiarity, word familiarity, and retention interval as within-subject factors. The Hit rate, FA rate and reaction times (RTs) were also analyzed by ANOVAs. Partial eta squared ($\eta_{p}^{2}$) was calculated to estimate the effect size of each analysis. *Post hoc* pairwise comparisons were Bonferroni-corrected (two-tailed, *p* < 0.05).

The recollection and familiarity processes were estimated using the independent K (IRK) procedure (Yonelinas, 2002; Yonelinas and Levy, 2002), in which R responses are assumed to estimate recollection, whereas familiarity is estimated as the proportion of K responses divided by the proportion of non-R responses. According to this procedure, the R and K responses are not only mutually exclusive, but they are also independently estimated. Then, the R and IRK responses were corrected using FA: Recollection = *p*(R, Hit)–*p*(R, FA); Familiarity = *p*(K, Hit)/(1 -*p*(R, Hit)) -*p*(K, FA)/(1-*p*(R, FA)). One subject’s data were excluded because he made all of the R/K/G judgments as K. Repeated measures ANOVA tests were performed separately for the recollection and familiarity processes with the retention interval, category familiarity, and word familiarity as within-subject factors.

## Results

The rating scores during encoding confirmed the results of word selection. The word rating scores were 4.19 ± 0.46 and 2.16 ± 0.46 for the familiar and unfamiliar words, respectively, *F*(1,20) = 326.96, *p* < 0.001, $\eta_{p}^{2}$ = 0.94. The words from the familiar categories were rated more familiar than those from the unfamiliar categories (3.56 ± 0.38 and 2.79 ± 0.42), *F*(1,20) = 179.76, *p* < 0.001, $\eta_{p}^{2}$ = 0.90. The interaction between category familiarity and word familiarity was significant, *F*(1,18) = 13.56, *p* < 0.001, $\eta_{p}^{2}$ = 0.40. Further analysis showed that the difference was larger for unfamiliar than familiar categories, although the difference in word familiarity was significant in both contrasts (*p*’s < 0.001) (**Figure 1C**). Note that the significant interaction may raise the possibility that the effect of category familiarity on unfamiliar words may be due to a larger difference in word familiarity. We performed further analysis (see details in the **Supplementary Material**) and ensured that the difference in word familiarity could not influence the effect of category familiarity in memory performance.

**FIGURE 3:**
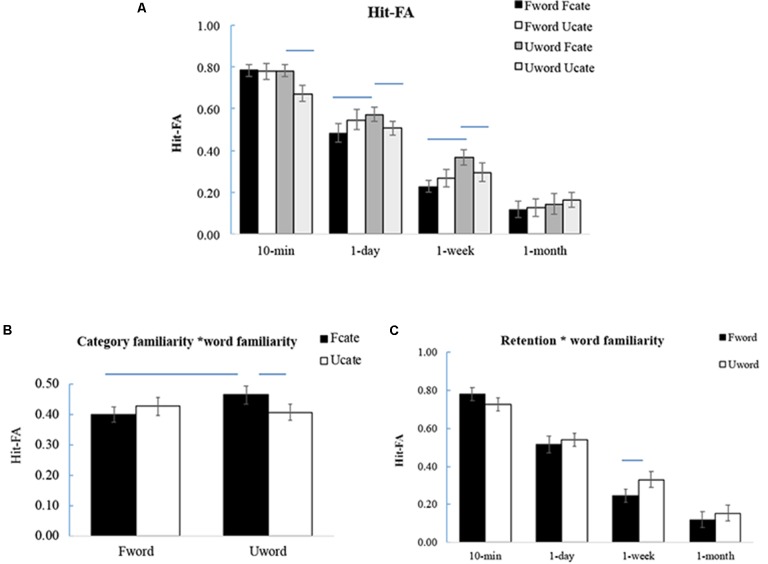
Results of the corrected recognition. **(A)** Corrected recognition at each time interval. **(B)** Interaction between category familiarity and word familiarity. The enhanced memory performance for Fcate vs. Ucate appeared for unfamiliar words but not for familiar words (UwordFcate > UwordUcate). In addition, unfamiliar words were recognized better than familiar words when the words were from a familiar category (UwordFcate > FwordFcate). **(C)** Interaction between retention interval and word familiarity. The unfamiliar words were recognized better than familiar words at longer intervals. The error bars represent the standard errors of the means. The lines above the bars represent significant difference between the two bars (*p* < 0.05). For **(A)**, only significant differences within each retention interval were labeled.

For the corrected recognition, the results showed that memory accuracy decreased over time [*F*(3,57) = 228.66, *p* < 0.001, $\eta_{p}^{2}$ = 0.92] (**Figure 3A**). There was a significant interaction between category familiarity and word familiarity [*F*(1,19) = 9.92, *p* = 0.005, $\eta_{p}^{2}$ = 0.34], and the interaction did not change with time [*F*(3,57) = 1.05, *p* = 0.38, $\eta_{p}^{2}$ = 0.05]. Further analysis showed that when the words were unfamiliar, memory performance was higher for the words with familiar categories than for those from unfamiliar categories (UwordFcate > UwordUcate, *p* = 0.02). When the words were familiar, there was a marginally significant effect of category familiarity with the opposite direction (FwordFcate < FwordUcate, *p* = 0.08) (**Figure 3B**). This suggested that category familiarity can promote memory performance for only the unfamiliar words. The interaction was also manifested as unfamiliar words were recognized better than familiar words when the words were from the familiar categories (UwordFcate > FwordFcate, *p* = 0.01).

Moreover, there was a significant interaction between retention interval and word familiarity [*F*(3,57) = 3.96, *p* = 0.012, $\eta_{p}^{2}$ = 0.17]. Further analysis showed that memory performance was higher for familiar words than for unfamiliar words at 10-min (*p* = 0.06); however, afterward, the pattern was opposite, especially at the 1-week interval (*p* = 0.02) (**Figure 3C**). This suggested that word familiarity enhances recent memory, but not remote memory. The effect of stimulus familiarity appeared at longer intervals. The values of corrected recognition under different conditions were significantly higher than expected by-chance (0) (*p*’s < 0.05).

**FIGURE 4:**
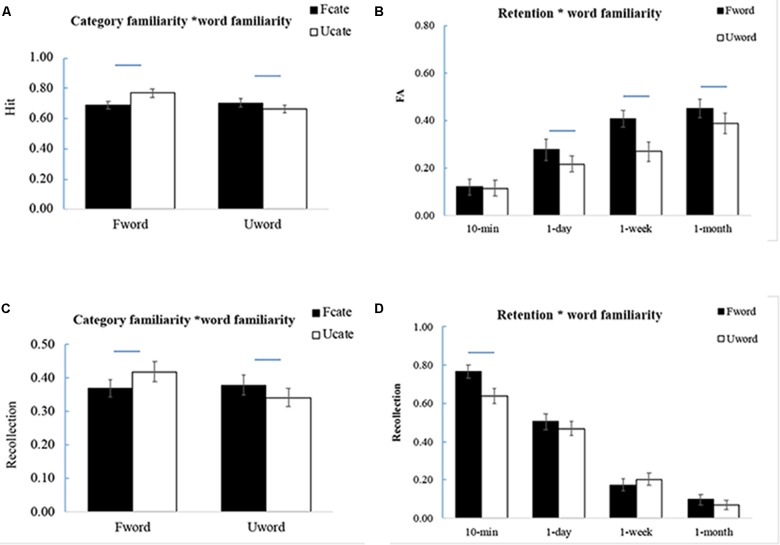
Interaction of category familiarity and word familiarity. **(A)** Interaction between category familiarity and word familiarity for the Hit rate. **(B)** Interaction between retention interval and word familiarity for the FA rate. **(C)** Interaction between category familiarity and word familiarity for recollection process. **(D)** Interaction between retention interval and word familiarity for recollection process. The error bars represent the standard errors of the means. The lines above the bars represent significant difference between the two bars (*p* < 0.05).

The Hit rate decreased significantly over time [*F*(3,57) = 37.62, *p* < 0.001, $\eta_{p}^{2}$ = 0.66] (**Table 1**). Similar to the results of corrected recognition, there was a significant interaction between category familiarity and word familiarity [*F*(1,19) = 27.97, *p* < 0.001, $\eta_{p}^{2}$ = 0.60]. Further analysis showed that the effect of category familiarity appeared for the unfamiliar words (UwordFcate > UwordUcate, *p* = 0.05), but in opposite direction for the familiar words (FwordFcate < FwordUcate, *p* < 0.001) (**Figure 4A**). The interaction between word familiarity and retention interval was not significant [*F*(3,57) = 0.31, *p* = 0.82, $\eta_{p}^{2}$ = 0.02]. It suggested that the enhanced effect of category familiarity for unfamiliar words is associated with higher Hit rate, as the participants correctly recognized more old words from familiar categories than from unfamiliar categories.

**Table 1:** Results in each experimental condition.

		FwordFcate	FwordUcate	UwordFcate	UwordUcate
Hit	10-min	0.89 ± 0.10	0.91 ± 0.08	0.88 ± 0.12	0.80 ± 0.14
	1-day	0.74 ± 0.13	0.84 ± 0.11	0.78 ± 0.16	0.73 ± 0.14
	1-week	0.59 ± 0.20	0.71 ± 0.14	0.61 ± 0.16	0.59 ± 0.20
	1-month	0.54 ± 0.22	0.60 ± 0.26	0.54 ± 0.22	0.54 ± 0.23
FA	10-min	0.11 ± 0.11	0.13 ± 0.15	0.10 ± 0.10	0.13 ± 0.12
	1-day	0.26 ± 0.18	0.30 ± 0.20	0.21 ± 0.14	0.23 ± 0.14
	1-week	0.36 ± 0.20	0.45 ± 0.23	0.24 ± 0.20	0.29 ± 0.19
	1-month	0.42 ± 0.25	0.48 ± 0.21	0.35 ± 0.21	0.38 ± 0.20
RTs (ms)	10-min	959 ± 108	967 ± 126	986 ± 146	999 ± 128
	1-day	985 ± 113	1015 ± 116	995 ± 102	993 ± 90
	1-week	988 ± 105	1023 ± 94	1006 ± 112	1000 ± 106
	1-month	1027 ± 135	1025 ± 137	1038 ± 150	1008 ± 90
Recollection	10-min	0.76 ± 0.16	0.78 ± 0.14	0.70 ± 0.16	0.58 ± 0.18
	1-day	0.47 ± 0.18	0.54 ± 0.18	0.50 ± 0.17	0.44 ± 0.15
	1-week	0.12 ± 0.14	0.23 ± 0.14	0.22 ± 0.13	0.18 ± 0.13
	1-month	0.09 ± 0.10	0.10 ± 0.10	0.08 ± 0.11	0.06 ± 0.09
Familiarity	10-min	0.32 ± 0.52	0.40 ± 0.47	0.42 ± 0.42	0.35 ± 0.33
	1-day	0.18 ± 0.19	0.23 ± 0.21	0.29 ± 0.26	0.14 ± 0.13
	1-week	0.13 ± 0.14	0.10 ± 0.16	0.16 ± 0.15	0.13 ± 0.13
	1-month	0.12 ± 0.13	0.10 ± 0.17	0.08 ± 0.10	0.09 ± 0.11


The false alarm (FA) rate increased significantly over time [*F*(3,57) = 34.31, *p* < 0.001, $\eta_{p}^{2}$ = 0.65] (**Table 1**). The FA was lower for the words from familiar categories than for those from unfamiliar categories [*F*(1,19) = 15.44, *p* = 0.001, $\eta_{p}^{2}$ = 0.45], which suggested that category familiarity enhanced the ability to distinguish the target from the distracters. The FA was higher for the familiar words than unfamiliar words [*F*(1,19) = 14.37, *p* = 0.001, $\eta_{p}^{2}$ = 0.43]. There was no significant interaction between category familiarity and word familiarity [*F*(1,19) = 2.86, *p* = 0.11, $\eta_{p}^{2}$ = 0.13]. The interaction between retention interval and word familiarity was significant [*F*(3,57) = 4.88, *p* = 0.004, $\eta_{p}^{2}$ = 0.20] because the FA of the familiar words was comparable to that of unfamiliar words at the 10-min interval (*p* = 0.79), but it was higher at the other intervals (*p*’s < 0.05) (**Figure 4B**).

For the RTs, there was no significant interaction between category familiarity and word familiarity [*F*(1,19) = 3.21 *p* = 0.09, $\eta_{p}^{2}$ = 0.17] (**Table 1**). The interaction between word familiarity and retention interval was not significant [*F*(3,57) = 1.91, *p* = 0.14, $\eta_{p}^{2}$ = 0.11].

Regarding the contribution of recollection, there was a significant effect of retention interval [*F*(3,54) = 236.77, *p* < 0.001, $\eta_{p}^{2}$ = 0.93] (**Table 1**). The interaction between category familiarity and word familiarity was significant [*F*(1,18) = 11.31, *p* = 0.003, $\eta_{p}^{2}$ = 0.39]. Same as that in corrected recognition, the effect of category familiarity appeared for unfamiliar words (UwordFcate > UwordUcate, *p* = 0.003). When the words were familiar, there was a significant effect of category familiarity in the opposite direction (FwordFcate < FwordUcate, *p* = 0.04) (**Figure 4C**). There also was a significant interaction between retention interval and word familiarity [*F*(3,54) = 5.92, *p* = 0.001, $\eta_{p}^{2}$ = 0.25]. Further analysis showed that the recollection estimate for familiar words was higher than that for unfamiliar words at 10-min (*p* = 0.003) but not at the other intervals (*p*’s > 0.15) (**Figure 4D**).

Regarding the contribution of familiarity, there was a significant effect of the time interval [*F*(3,54) = 8.60, *p* < 0.001, $\eta_{p}^{2}$ = 0.31] (**Table 1**). The interaction between category familiarity and word familiarity [*F*(1,18) = 2.19, *p* = 0.16, $\eta_{p}^{2}$ = 0.10], between retention interval and word familiarity [*F*(3,54) = 0.22, *p* = 0.82, $\eta_{p}^{2}$ = 0.01] were not significant.

## Discussion

In this study, we explored the extent to which category familiarity and word familiarity interacted to influence subsequent memory performance. There were two main findings. First, there was a significant interaction between category familiarity and word familiarity for memory accuracy. The interaction manifested in two aspects. One was that the enhancing effect of category familiarity was shown for unfamiliar words. That is, prior conceptual knowledge promoted memory performance for acquiring new stimuli. When the words were familiar to participants, words that did not involve category familiarity were remembered better than those involving category familiarity. The other was that within the familiar category, unfamiliar words were recognized better than familiar words. That is, the effect of the stimulus familiarity only appeared within the existing conceptual systems. Second, there was a significant interaction between word familiarity and retention interval. At 10-min, familiar words were remembered better than unfamiliar words, but over time, the opposite pattern occurred. These results clarified the boundary conditions under which prior conceptual knowledge facilitates or interferes with memory processes.

### Enhancing Effect of Prior Conceptual Knowledge for Unfamiliar Words

The results clarified the boundary condition for the effects of category familiarity and stimulus familiarity. Previous studies have shown that prior knowledge has both enhancing and detrimental effects on memory performance. By separating category familiarity and stimulus familiarity, we found that the effect of category familiarity enhanced subsequent memory only for unfamiliar words, but not for familiar words.

The higher memory performance for unfamiliar words was mainly due to a higher Hit rate and was mainly driven by the recollection process. Previous studies have also shown that the effect of category familiarity is recollection based (e.g., Brandt et al., 2005; Toth et al., 2011; DeWitt et al., 2012). This suggested that category familiarity increases the availability of details that can support later recollection. Prior category knowledge can free attention-related resources and allocate them to encoding of the feature details that are associated with the prior knowledge (Craik and Tulving, 1975; Rawson and Van Overschelde, 2008; DeWitt et al., 2012). During retrieval, the participants can use the information in their prior category knowledge system to aid memory by retrieving information and associations made during encoding. All these processes facilitate assimilation of new stimuli into a preexisting knowledge system (DeWitt et al., 2012; Van Kesteren et al., 2012). Note that unlike some previous studies that enrolled participants with different knowledge backgrounds (e.g., Long and Prat, 2002; Brandt et al., 2005; Van Kesteren et al., 2014), more than one familiar and unfamiliar category was included in this study. As the current results were consistent with previous findings, they suggested that the underlying mechanisms are the same, and the effect of prior conceptual knowledge can be applied to more general situations.

For the familiar words, there was higher memory performance for unfamiliar categories than for familiar categories, showing an incongruent effect (e.g., Neuschatz et al., 2002; Davis et al., 2012). This effect was mainly due to higher Hit rate and FA rate. Prior conceptual knowledge enables participants to make more accurate and more confident judgments, but it can also induce a higher FA rate (Neuschatz et al., 2002; Bird et al., 2011; De Brigard et al., 2017). This suggested that familiar words within the familiar categories are more easily interfered with other words within familiar categories. In contrast, for familiar words in unfamiliar categories, there is less interference and these words are more distinctive.

### Effect of Stimulus Familiarity Within the Prior Conceptual Knowledge

The effect of stimulus familiarity refers to enhanced memory for unfamiliar than familiar words. The results clarified that the effect of stimulus familiarity only appeared for words from familiar categories, but not from unfamiliar categories. The effect of stimulus familiarity has been verified in recognition tests in many studies (e.g., Deese, 1959; Gorman, 1961; Rao and Proctor, 1984; Kumaran and Maguire, 2007). This is mainly because when a network of prior conceptual knowledge is activated, familiar words are subject to more interference than unfamiliar words (Anderson, 1983). During retrieval, the rejection of familiar words requires better discrimination between old items and familiar items from the existing systems as opposed to rejection of unfamiliar words by the absence of item familiarity alone (Poppenk et al., 2010). Different from the effect of category familiarity, the effect of stimulus familiarity was related to a lower FA rate for unfamiliar than familiar words. The old and new words were harder to distinguish when they were familiar in both category and stimulus ratings (i.e., FwordFcate).

This was consistent with the studies that demonstrated false memories when individuals incorrectly identify new, but related, information as old, and higher false memory for highly schematic lures (Webb et al., 2016; for review, see Koriat et al., 2000). Note that these false memories usually appeared within the prior conceptual knowledge system (Poppenk et al., 2010), or familiar episodic events (e.g., lectures, bathroom, classroom) (e.g., Neuschatz et al., 2002; Yamada and Itsukushima, 2013). In these studies, lure items that are similar to the old items have higher FA in schematic than non-schematic information. We also found that familiar words have higher FA than unfamiliar words. This suggests that within the prior conceptual knowledge, the ability to discriminate old and new words is better for unfamiliar words, as they are subject to less interferences, and false memory may occur because of high interference from detailed memory. It is possible that prior conceptual knowledge helps one to obtain detailed information for unfamiliar words, thus facilitating their memory performance.

### Interaction Between Word Familiarity and Retention Interval

Previous studies have suggested that information related to prior conceptual knowledge is consolidated more quickly (Tse et al., 2007; Takashima et al., 2009; Lewis and Durrant, 2011). Our results provided evidence that the effect of category familiarity occurred immediately after encoding, and once it was established, it remained stable over time. Thus, although memory performance decreased over time, the effects of category familiarity did not change.

Differently, there were significant interactions between the time interval and word familiarity for the corrected recognition, FA rate, and recollection estimates. Memory performance was higher for familiar than unfamiliar words only at the 10-min interval, and the results depended on the recollection process. Over time, the recollection process decayed (Sadeh et al., 2014) and the advantage for familiar words disappeared, which led to the opposite pattern. This suggested that, different from the effect of category familiarity, the effect of stimulus familiarity is more likely to be observed at longer intervals, rather than minutes after exposure. This was consistent with models that assume that unfamiliar words are more distinctive than familiar words (e.g., Neuschatz et al., 2002; De Brigard et al., 2017). The FA results confirmed that there was a higher FA rate for familiar than unfamiliar words, that is, participants had greater difficulty in distinguishing old words from their distractors when words were familiar.

### Limitations and Future Directions

There are some potential directions for future investigations. First, in this study, different groups of participants performed the category familiarity rating and memory tasks. Further studies are needed to define category familiarity based on the same group of participants. Second, word familiarity is one feature of the words. Words also differ in other measures, such as word typicality and word imageability. These factors are important in lexical-semantic processing. Studies have shown that word familiarity is positively correlated with other measures, such as familiarity, typicality, complexity, imageability, and age of acquisition (Morrow and Duffy, 2005). However, whether other measures influence the effect of category familiarity, needs further investigation. Third, in the word familiarity rating, to get enough number of trials for four retention intervals, we did not match the category difference in word ratings, which raised the possibility that the effect of category familiarity in unfamiliar words may be due to a larger difference in word familiarity. Although we matched the word familiarity for familiar and unfamiliar categories by dropping some trials in the **Supplementary Material**, and found similar results in memory performance, it would be best to manipulate word familiarity and category familiarity completely orthogonally in future studies.

## Conclusion

In summary, the interaction of category familiarity and word familiarity was manifested in two aspects. One was that the presence of familiar categories enhanced memory for unfamiliar words but not for familiar words. The other was that within the familiar categories, unfamiliar words were recognized better than familiar words, showing effect of stimulus familiarity. The recollection process contributed to the effect of category familiarity. This study dissociated the familiarity of category and stimulus and clarified the boundary condition for the effects of prior knowledge on memory enhancement.

## Ethics Statement

The study was approved by the ethics committee of School of Psychological and Cognitive Sciences, Peking University. Participants received written and oral information of the study before they gave their written consent.

## Author Contributions

XN designed the research, performed the research, and analyzed the data. CL analyzed the data. JY designed the research, analyzed the data, and wrote the paper.

## Conflict of Interest Statement

The authors declare that the research was conducted in the absence of any commercial or financial relationships that could be construed as a potential conflict of interest.
