# Supplementary material for: Word Familiarity Modulated the Effects of Category Familiarity on Memory Performance
Source: Front Psychol. 2018 Aug 8;9:1429. doi: 10.3389/fpsyg.2018.01429 (PMC6092683; doi:10.3389/fpsyg.2018.01429)
Supplement: Supplementary file 1 [file Data_Sheet_1.docx]

# SUPPLEMENTARY MATERIAL

In the word familiarity rating, there was a significant interaction between category familiarity and word familiarity, as the effect of word familiarity was larger for the unfamiliar category than for the familiar category. In another aspect, the category difference was larger for unfamiliar words than for the familiar words in word ratings (Figure 3a). This raised the possibility that the effect of prior knowledge in unfamiliar words may be due to a larger difference in word familiarity.

To address this possibility, we adopted two approaches. For the first approach, we matched the category difference for familiar and unfamiliar words in word ratings, by dropping trials of the highest word ratings for UwordFcate and trials of the lowest word ratings for UwordUcate. The reason why we selected to drop the trials of the two conditions was that it was the way that we could drop the fewest number of trials in total to eliminate the interaction between category familiarity and word familiarity. The remaining number of trials of UwordFcate was 101 (out of 144), and that of UwordUcate was 104 (out of 144). In the rating results, there was no significant interaction between word familiarity and category familiarity, *F*(1,20) = 0.53, *p =* 0.48, η_p_^2^ = 0.03, while the main effects of word familiarity (*F*(1,20) = 251.76, *p <* 0.001, η_p_^2^ = 0.93) and of category familiarity (*F*(1,20) = 122.89, *p <* 0.001, η_p_^2^ = 0.86) were significant (suppl. Figure 1a). The analysis of corrected recognition showed similar results to those including all trials (Figure 3). There was a significant interaction between word familiarity and category familiarity (*F*(1,19) = 6.50, *p =* 0.02, η_p_^2^ = 0.26), and a significant interaction between word familiarity and retention interval (*F*(1,19) = 4.35, *p =* 0.01, η_p_^2^ = 0.19) (suppl. Figure 1b).

For the second approach, we further diminished the category difference for familiar and unfamiliar words in word ratings. We dropped trials of the highest word ratings for familiar categories and trials of the lowest word ratings for unfamiliar categories. The criterion was the same as the first approach, i.e., dropping as few number of trials in total as possible, when both main effect of category familiarity and the interaction were eliminated. The remaining number of trials was as following: FwordFcate as 64, UwordFcate as 70, FwordUcate as 62, and UwordUcate as 71. In the rating results, there was neither a significant interaction between word familiarity and category familiarity *F*(1,20) = 0.30, *p =* 0.15, η_p_^2^ = 0.10, nor a significant main effect of category familiarity (*F*(1,20) = 0.37, *p =* 0.55, η_p_^2^ = 0.02). The effect of word familiarity was significant (*F*(1,20) = 192.60, *p <* 0.001, η_p_^2^ = 0.91) (suppl. Figure 1c). Similar to the results those including all trials (Figure 3), the corrected recognition showed a significant interaction between word familiarity and category familiarity (*F*(1,19) = 3.26, *p =* 0.07, η_p_^2^ = 0.15), and a significant interaction between word familiarity and retention interval (*F*(1,19) = 4.05, *p =* 0.01, η_p_^2^ = 0.18) (suppl. Figure 1d).

The results obtained in both approaches suggested that the difference in word familiarity should not influence the effect of category familiarity in memory performance.

# FIGURE LEGENDS

Supple Figure 1. Results for excluding category difference in word ratings. A: Word familiarity rating during encoding after excluding the interaction of word familiarity and category familiarity. B: Corrected recognition performance for approach 1. C: Word familiarity rating during encoding after further excluding the effect of category familiarity. D: Corrected recognition performance for approach 2. The lines above the bars represent significant difference between the two bars (*p* <0.05). For Figure b and d, only the significant differences within each retention interval were labeled.
